# AIS Trajectories Simplification Algorithm Considering Topographic Information

**DOI:** 10.3390/s22187036

**Published:** 2022-09-17

**Authors:** Wonhee Lee, Sung-Won Cho

**Affiliations:** Maritime Safety and Environmental Research Division, Korea Research Institute of Ships and Ocean Engineering, 32, Yuseong-daero 1312 beon-gil, Yuseong-gu, Daejeon 34103, Korea

**Keywords:** AIS information, PMR quadtree, Douglas–Peucker algorithm, trajectories simplification, topographic information

## Abstract

With the development of maritime technology and equipment, most ships are equipped with an automatic identification system (AIS) to store navigation information. Over time, the size of the data increases, rendering its storage and processing difficult. Hence, it is necessary to transform the AIS data into trajectories, and then simplify the AIS trajectories to remove unnecessary information that is not related to route shape. Moreover, topographic information must be considered because otherwise, the simplified trajectory can intersect obstacles. In this study, we propose an AIS trajectory simplification algorithm considering topographic information. The proposed algorithm simplifies the trajectories without the intersection of the trajectory and obstacle using the improved Douglas–Peucker algorithm. Polygon map random (PMR) quadtree was used to consider topographic information on the coast, and the intersection between topographic information and simplified trajectories was efficiently computed using the PMR quadtree. To verify the effectiveness of the proposed algorithm, experiments were conducted on real-world trajectories in the Korean sea. The proposed algorithm yielded simplified trajectories with no intersections of the trajectory and obstacle. In addition, the computational efficiency of the proposed algorithm with the PMR quadtree was superior to that without the PMR quadtree.

## 1. Introduction

The International Maritime Organization (IMO) adopted the introduction of automatic identification systems (AISs) to enhance the navigation safety of ships and protection of the marine environment [1]. An AIS is a device that automatically transmits and receives data between ships and AIS base stations, including static information (such as Maritime Mobile Service Identity (MMSI) number, call sign, type, and length), dynamic information (such as position, time, speed over ground, and navigational status), voyage-related information, and short safety messaging [2]. AIS data can be used to generate traffic networks [3,4], predict ship behavior [5,6,7,8] and trajectory [9,10], support maritime search and rescue systems [11,12], detect fishing activity [13], create networks [14] and routes [15] for navigation, and detect abnormal behavior [16,17,18,19].

In previous studies, knowledge was extracted from AIS data, and researchers commonly emphasized the importance of data preprocessing. There are challenges in extracting knowledge from AIS data because it has limitations such as sensor error, volume of data, incompleteness, and noise [2,20,21,22,23]. Among these preprocessing techniques, reducing the number of AIS-data points to reduce storage and computation costs is significant. AIS data are stored for periods of 5–100 s on the coast, and more than 100 million data points are generated every day within areas with high ship traffic. Eliminating unnecessary data points without losing information on the data has been studied in two directions: trajectory simplification and event identification [18]. Trajectory simplification eliminates redundant data points for representing the shapes while maintaining the overall shape of the trajectory [24]. Event identification involves analyzing AIS data for identifying the ship’s arrival and departure from ports, stop, and turn, and extracting the desired trajectory [25,26,27,28,29,30]. In this study, we focused on trajectory simplification.

AIS trajectories simplification has been studied using line simplification methodology [31]. Shi et al. [32], Ji et al. [33], and Qi and Ji [34] compared various algorithms for simplifying AIS trajectories, including the choosing interval points, limiting vertical distance, limiting angle, offset angle, grating, Douglas–Peucker (DP) [35], Opheim [36], and Visvalingam–Whyatt algorithms [37]. Among these, the DP algorithm has been demonstrated to have fewer errors and better performance compared to the others.

Most studies on AIS trajectories simplification are based on the DP algorithm [38]. In the DP algorithm, the threshold is the sole parameter that determines the shape of the simplified trajectory. Therefore, methods for determining the threshold have generally been studied. Etienne et al. [39] proposed a spatio-temporal DP algorithm to simplify the trajectories and reduce the computation time. Muckell et al. [40] proposed an extended spatial quality simplification heuristic (SQUISH-E) algorithm. The SQUISH-E algorithm simplifies the trajectory by considering the errors caused by AIS data removal. Zhang et al. [31] determined the threshold of the DP algorithm using the size of the minimum ship domain as the evaluation criterion. Li et al. [41] determined the threshold of the DP algorithm through numerous experiments to ensure a good balance between AIS trajectory simplification and visualization performance. Singh et al. [42] developed the scan–pick–move (SPM) algorithm based on the DP algorithm. The SPM algorithm has lower computational complexity compared to the DP algorithm. Zhang et al. [43] conducted a study to set the threshold value on the basis of ship specifications. Zhao and Shi [44] extracted the data points at which the angle changes in the trajectory using the course over ground (COG) information from the AIS data. Huang et al. [45] proposed a GPU-based parallelization framework for shortening the computation time of the DP and the kernel density estimation algorithms for trajectory compression and visualization. Wei et al. [46] simplified the trajectories by combining the sliding window technique that simplifies the speed and course data with the DP algorithm. They used a threshold of 0.8 times the length of the ship, as proposed by Zhang et al. [31].

Many studies have set a static value as a threshold, which causes the poor compression quality of the trajectory. Recently, an adaptive threshold method responding to the condition of each trajectory has been studied to solve this problem. Liu et al. [47] proposed an algorithm that automatically generates the threshold value of the DP algorithm according to the average distance of the trajectory. Tang et al. [48] proposed an algorithm that identifies the turning points that rotate more than a certain angle and used them to change the threshold. Ji et al. [49] proposed an adaptive grating algorithm that can dynamically generate an appropriate threshold for each trajectory.

Although many studies have proposed AIS trajectories simplification methods based on the DP algorithm, no studies have considered topographic information. The reasons for not considering the information are twofold: First, there was no need to consider topographic information because it simplified the trajectory at sea without islands or obstacles. Second, the threshold of the DP algorithm was set to be small, and the number of simplifications was minimized. Since the AIS data represent the locations that the ship passed, a simplified trajectory to avoid obstacles can be generated if the threshold is set small even without considering the topographic information. However, depending on the user-defined threshold of the trajectory simplification algorithm, the intersection of the trajectory and obstacle occurs, or many unnecessary data points remain because the trajectory is not sufficiently simplified. Therefore, we propose the obstacle Douglas–Peucker (ODP) algorithm that considers topographic information.

In the ODP algorithm, obstacle detection is combined with the DP algorithm. To detect obstacles, the intersection of line segments and polygon (ILP) algorithm is used to determine the intersection of a trajectory and obstacle because topographic information and trajectories are represented by polygons and lines, respectively. The DP algorithm has a recursive characteristic to repeatedly perform simplification. Thus, in areas such as coastal areas with many obstacles, the ILP algorithm further increases the computational burden. To address this challenge, we utilize a grid-based technique. Typically, grid-based methods, such as the K-dimensional tree, binary tree, quadtree, octal tree (octree), and R-tree, are used to represent complex obstacle fields [50,51]. The size of the grid must be significantly small to represent complex marine environments with the binary or K-dimensional tree [52]. However, these methods generate inefficient data storage, representing all areas with redundant information in a large terrain. In particular, this is critical because the sea, which contains large land masses over several kilometers, is the scope of the study. The quadtree is widely used in the modeling of complex and large-scale environments such as land [53,54], sea [55,56,57], earthquakes ground motion [58], flood [59], and tsunami [60]. Therefore, we utilized a quadtree as the spatial data structure to efficiently store topographic information and represent the free space.

The contributions of this study are summarized as follows: (1) A novel algorithm that simplifies the AIS trajectories considering topographic information is proposed. (2) To perform the ODP algorithm in a time frame, the ILP algorithm with the polygon map random (PMR) quadtree is proposed. (3) The practical restrictions for navigation on the coast are considered, and the effectiveness is demonstrated through testing in real-world environments.

The remainder of this paper is organized as follows. In Section 2, an AIS trajectory simplification algorithm considering topographic information is proposed. In Section 3, the results are confirmed and analyzed by applying the proposed algorithm to AIS trajectories. Finally, the conclusions and future plans are discussed in Section 4.

## 2. Methods

The ODP algorithm comprises three phases: (1) data preprocessing that removes outliers among the AIS data points and generates trajectories; (2) detecting the intersection of line segments and the polygon using the PMR quadtree; (3) simplifying the AIS trajectories using the DP algorithm considering topographic information.

### 2.1. AIS Data Preprocessing

Raw AIS data are difficult to use for analysis because the information from several ships is mixed. Therefore, data classification is essential. AIS data contain static information, such as MMSI number, IMO number, ship name, ship type, length, width, draft, and dynamic information, such as latitude, longitude, speed, COG, speed over ground (SOG), ship heading, and date (format: yyyy-mm-dd hh:mm:ss). Using the MMSI number, the ship data were classified using the MMSI. Then, each ship data was divided based on the berthing or anchoring, which means the ship was stopped for a certain period; this is called a voyage.

For each voyage data, preprocessing was performed. First, AIS data were chronologically sorted to detect outliers, which means abnormal location information caused by a system malfunction or data transmission error. Subsequently, data with SOG of three knots or less were removed owing to the characteristics of the ship. When the ship is berthing or anchoring, the SOG is nearly zero. Because the voyage with removed data did not equal time intervals, it needed to be set with the same time interval. To have the same time interval for the routes, resampling was performed. The resampling criterion was the AIS data time interval. In this study, the time interval was set to 1 s, which is the minimum time difference between two consecutive data points.

### 2.2. ILP Algorithm Using Quadtree

We propose an ILP algorithm that efficiently reduces the computational burden by using the PMR quadtree. A simple method to check the intersection of line segments and polygons is to determine whether they are crossed for all line segments. Because this method is time-consuming and inefficient, previous studies have proposed methods to improve it; however, the proposed methods were difficult to apply for spatial data of significantly large sizes [52,53,54,55,56,57,58,59,60,61]. Therefore, in this study, we propose an efficient ILP algorithm using the PMR quadtree.

The tree is a data structure in which each internal node has a group of several sub-nodes. Since the number of sub-nodes is set to the powers of 2 (2, 4, 8…), they are referred to as a binary tree, quadtree, octal tree (octree), and so on. Among these trees, the quadtree is widely used for topographic visualization and spatial processing. There are various types of quadtrees depending on the input data format and splitting method. Generally, there is a point region (PR) quadtree, used when the input data are points, and a polygon map (PM) quadtree, used when the input data are lines. Since topographic information is polygonal information composed of lines, a PM quadtree is suitable. However, the PM quadtree has the limitation of being time-consuming for the quadtree creation because points are included in the split conditions. To overcome this limitation, a PMR quadtree that sets the splitting condition as a line is proposed. Because the PMR quadtree subdivides the tree by inserting the input data one by one, the tree structure is rapidly created. Using PMR quadtree, the execution time per input data is similar as the size of the input data increases. Moreover, the CPU cost is smaller than that of other tree methods. In the previous study, it was confirmed that the PMR quadtree yielded results similar to the those of other tree methods, while producing the results at least five times faster compared to other tree methods [62].

**Figure 1:**
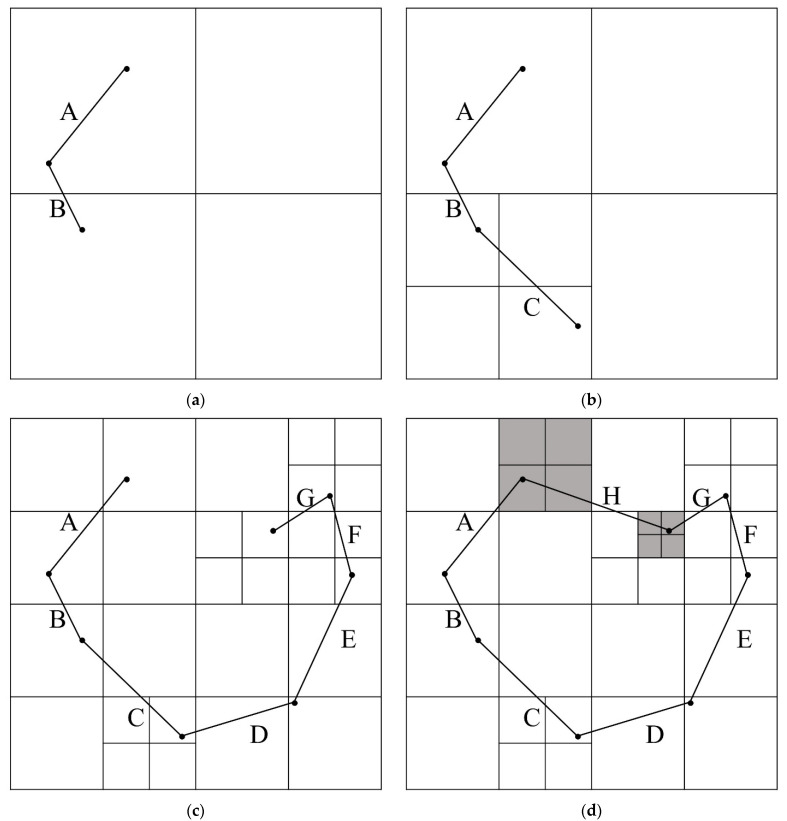
Example of polygon map random (PMR) quadtree generation: (**a**) Quadtree generated by line segment B; (**b**) Quadtree generated by line segment C; (**c**) Quadtree generated by line segment D–G; (**d**) Quadtree generated by line segment H.

The PMR quadtree checks the condition of the node related to line segments constituting the topographic information and divides the existing node into four sub-nodes to generate a grid map. The hyperparameters of the PMR quadtree are the splitting threshold and maximum depth. The splitting threshold is the maximum number of line segments required to divide the current node into sub-nodes; the maximum depth is a parameter for limiting the number of divisions of the initial node. When a line segment is input, if the related node exceeds the splitting threshold and the depth of the node does not exceed the maximum depth, the node is split into four sub-nodes. A node that is no longer divided is called a leaf node. Figure 1 depicts an example of creating a PMR quadtree, where the splitting threshold was set to 1 and the maximum depth was set to 4. The line segments constituting the polygon were input in the order of A–H. The initial node exceeds the splitting threshold of 1 when line segment B is input. As shown in Figure 1a, the initial node is split into northeast (NE), northwest (NW), southeast (SE), and southwest (SW) nodes. When line segment C is input, the number of line segments in the SW node exceeds the splitting threshold. Therefore, it is divided as shown in Figure 1b. Following this method, the line segments D–G generate the quadtree, as shown in Figure 1c, and the line segment H divides the nodes in Figure 1c as shown in the gray areas in Figure 1d.

**Figure 2:**
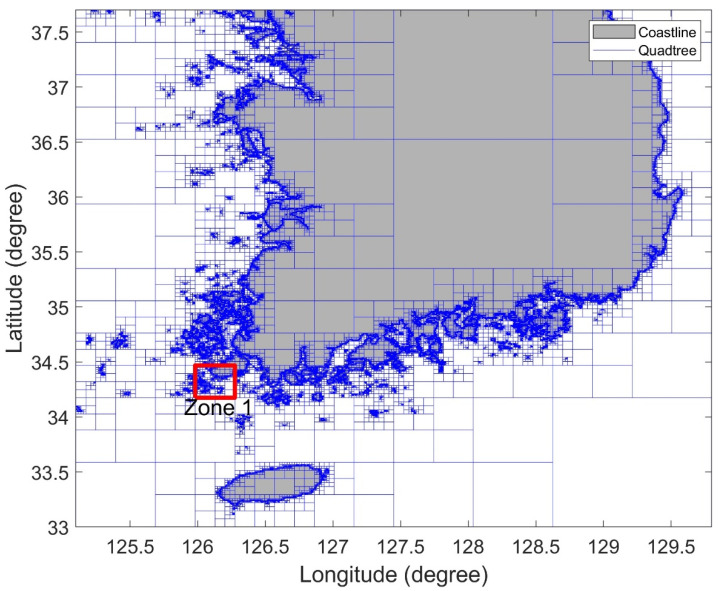
PMR quadtree visualization in Korean sea.

**Figure 3:**
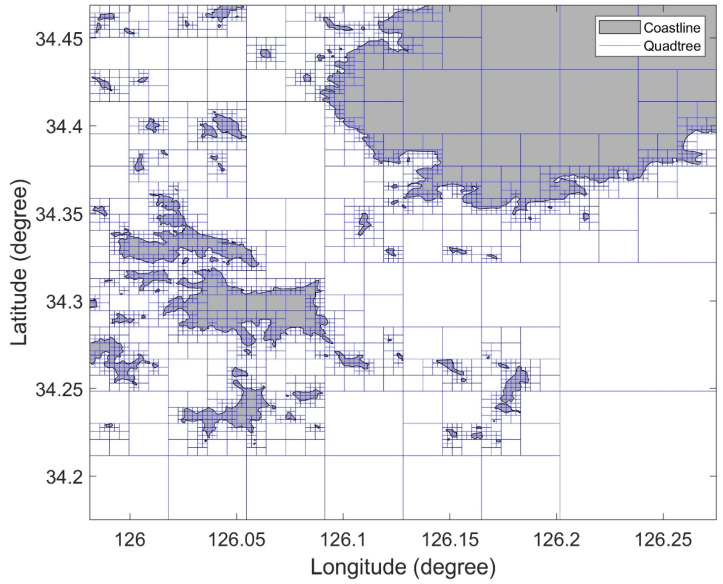
Detailed view of Zone 1 in Figure 2.

The topographic information of the Korean sea consists of the coastline and more than 2500 islands. Figure 2 shows the Korean sea expressed using the PMR quadtree. The longitudinal and latitudinal boundaries of the quadtree were set as 125.1°–129.8° E and 33°–37.7° N, respectively. The splitting threshold was set to 2, and the maximum depth to 12. The area marked in Figure 2 is enlarged as shown in Figure 3.

The line segments of topographic information overlapping boundaries are stored in the leaf node of the PMR quadtree. By using this characteristic, the intersection of a trajectory and an obstacle can be determined.

The overall procedure of the ILP algorithm using quadtree is described by Algorithm 1.
**Algorithm 1** Edge acquisition in quads intersecting with lineInput: root,p,q
Output: edgeSet1:**function** LineIntersectPoly(p,q)2: GetEdges(node,p,q,edgeSet)
3: **for**
i = 1 to N  //N is the number of edges in edgeSet
4:  **If**
edgeSet[i] $\cap^{\;}$ (p,q) **then**
**return** true5: 
**end for**
6: **return** false7:**end function**8:**function** GetEdges(node,p,q,edgeSet)
9: **if**
node.child ≠∅
**and**
node.edges ≠∅
**then**10:  edgeSet.append(node.edges) 11: 
**else**
12:  child = {NE, NW, SE, SW}13:  **for**
*i* = 1:4 **do**14:   **if** node.child[i].bound $\cap^{\;}$ (p,q) **then**15:    GetEdges(node.child[i],p,q,edgeSet)
16:   
**end if**
17:  
**end for**
18: 
**end if**
19:**end function**

**Figure 4:**
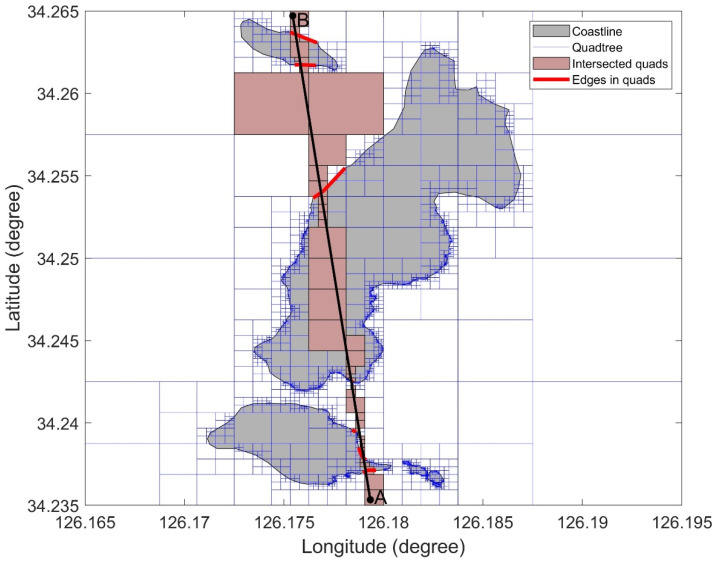
Example of edges in quads intersecting with line.

An example of Algorithm 1 is shown in Figure 4. Figure 4 contains topographic information within the longitudinal and latitudinal boundaries of 126.165°–126.195° N and 34.235°–34.265° E, respectively. The total number of line segments was 600. Line segments A (126.1793° N and 34.2353° E)–B (126.1754° N and 34.2647° E) were provided. The boundaries of the quadtree (intersected quads) intersecting with the line segment A–B are shown as the region shaded in pink in Figure 4. The topographic edges contained in the quadtree intersecting with line segments A–B are shown in red. The ILP algorithm using quadtree reduced the number of line segments for checking intersections from 600 to 13. The proposed algorithm drastically improved efficiency. Subsequently, the proposed algorithm was used for the trajectory simplification algorithm considering the topographic information.

### 2.3. Trajectory Simplification Algorithm Considering Topographic Information

The DP algorithm is a method for approximating an existing shape to a line, which requires a threshold to simplify the line [35]. The existing DP algorithm simplifies the trajectory without considering the topographic information. However, if there are complicated coastlines or islands, topographic information must be considered. When the threshold is small, there is no issue with simplification. However, as the threshold increases, the trajectory is simplified as it intersects the obstacle. Therefore, we propose the DP algorithm that considers the topographic information, as shown in Algorithm 2.
**Algorithm 2** ODP algorithmInput: trj(trajectory), eps(threshold)Output: s_trj(simplified trajectory)1: **function** Obstacle_DP(trj,eps,s_trj)
2:  n = trj.size()3:  index, $d_{max}$ = PerpendicularGeoDistance (trj, trj[1],trj[n])4:  **if** $d_{max}>eps$ or LineIntersectPoly (trj[1],trj[n])
5:   result1 = Obstacle_DP (trj[1],trj[1]+index)
6:   result2 = Obstacle_DP (trj[1]+index,trj[n])
7:   *s_trj* = [result1 result2]8:  
**else**
9:   *s_trj* = [trj[1] trj[n]]10:  
**end if**
11:**end function**

**Figure 5:**
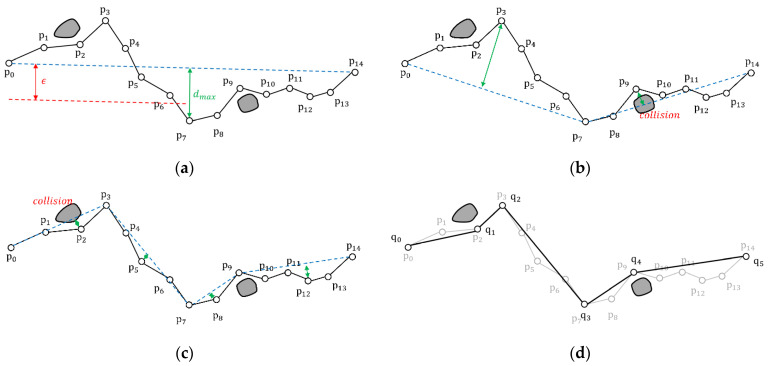
Route simplification using the ODP algorithm: (**a**) Check the original trajectory $p_{0}$–$p_{14}$; (**b**) Check the subtrajectories $p_{0}$–$p_{7}$ and $p_{7}$–$p_{14}$; (**c**) Check the subtrajectories $p_{0}$–$p_{3}$, $p_{3}$–$p_{7}$, $p_{7}$–$p_{9}$, and $p_{9}$–$p_{14}$; (**d**) Simplified trajectory $q_{0}$–$q_{5}$ simplified from the original trajectory (grey).

The ODP algorithm proceeds as shown in Figure 5. As shown in Figure 5a, the distance between the generated line segment, connecting $p_{0}$ and $p_{14}$ (the blue dashed line), and the point $p_{7}$, which had the largest vertical distance ($d_{max}$) was compared with the threshold (ϵ). Because it is greater than ϵ, $p_{7}$ was fixed and two subtrajectories ($p_{0}$–$p_{7}$ and $p_{7}$–$p_{14}$) were generated as shown in Figure 5b. Figure 5b illustrates that, in the trajectory from $p_{0}$ to $p_{7}$, the maximum vertical distance was greater than ϵ; therefore, the trajectory was divided into two subtrajectories ($p_{0}$–$p_{3}$ and $p_{3}$–$p_{7}$). In the trajectory from $p_{7}$ to $p_{14}$, the maximum vertical distance was smaller than ϵ. However, the trajectory was divided into two subtrajectories ($p_{7}$–$p_{9}$ and $p_{9}$–$p_{14}$) owing to the obstacle. Furthermore, in Figure 5c, it can be observed that the trajectory from $p_{0}$ to $p_{3}$ intersects with the obstacle; therefore, it was divided into two subtrajectories ($p_{0}$–$p_{2}$ and $p_{2}$–$p_{3}$). In other trajectories, the maximum vertical distance was smaller than ϵ, and no intersection with the obstacle could occur; therefore, the trajectories are simplified. Finally, as shown in Figure 5d, the trajectory from $p_{0}$ to $p_{14}$ was simplified to the trajectory from $q_{0}$ to $q_{5}$. A simplified trajectory does not intersect with the obstacle.

**Figure 6:**
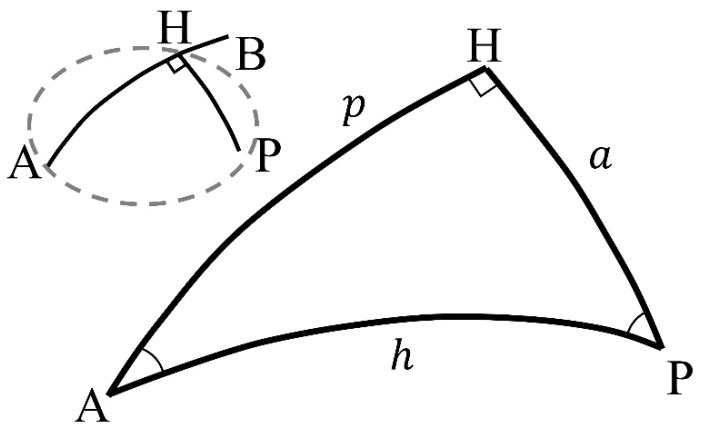
Spherical triangle for perpendicular distance.

In the ODP algorithm (4th line in Algorithm 2), the perpendicular distance of a point from a straight line on Earth is computed. As shown in Figure 6, a triangle comprising points A, P, and H on the Earth is called a spherical triangle. In Equations (1) and (2), *a* and *h* are distances (km); A and H are angles (rad); $l_{PH}$ and $l_{AP}$ are the distances (km) between points P and H, and points A and P, respectively; R is Earth’s radius (km); $\theta_{AH}$ and $\theta_{AP}$ are the azimuth angles (rad) between points P and H, and points A and P, respectively. The sine rule of a spherical triangle is formulated as Equation (1) [63]. Because the angle H is 90°, sinH=1, and it can be written as Equation (2) considering the unit of distance. The angle A is computed using the difference between the azimuth angles $\theta_{AH}$ and $\theta_{AP}$.

(1) sinasinH=sinhsinA

(2) $$l_{PH}=R\cdot \mathrm{arc}\sin\left(\sin\frac{l_{AP}}{R}\right)\sin\left(\theta_{AH}-\theta_{AP}\right)$$

In Equations (3) and (4), θ is the azimuth (rad), l is the distance (km), ϕ is the longitude, λ is the latitude, and Δλ is the latitudinal difference between the two points. Equation (3) is used for calculating the azimuth, and Equation (4) is the haversine distance equation for calculating the distance between two points on Earth.

(3) $$\theta =\mathrm{arc}\tan2\left(\sin\Delta \lambda \cos\phi_{2},\cos\phi_{1}\sin\phi_{2}-\sin\phi_{1}\cos\phi_{2}\right)\Delta \lambda$$

(4) $$l=2r\cdot \mathrm{arc}\sin\left(\sqrt{\sin^{2}\left(\frac{\phi_{2}-\phi_{1}}{2}\right)+\cos\phi_{1}\cos\phi_{2}\sin^{2}\left(\frac{\lambda_{2}-\lambda_{1}}{2}\right)}\;\right)$$

### 2.4. Evaluation Metric

A trajectory can be evaluated using length loss, compression rate, and violations. The length loss can be computed using the length of the original trajectory and the simplified trajectory according to Equations (5) and (6).

(5) $$L=\overset{N-1}{\underset{n=1}{\sum }}d_{n},$$

(6) $$LengthLoss=\left(1-\frac{L_{S}}{L_{o}}\right)\cdot 100,$$

where $d_{n}$ denotes the haversine distance between two adjacent points on one trajectory. L is the total length of the trajectory. $L_{O}$ is the total length of the original trajectory, and $L_{S}$ is the total length of the simplified trajectory. The length loss can be computed using the ratio of $L_{O}$ and $L_{S}$.

The compression rate denotes how many points on the original trajectory were removed and can be expressed as Equation (7).

(7) $$CompressionRate=\left(1-\frac{N_{S}}{N_{o}}\right)\cdot 100,\;$$

where $N_{O}$ denotes the number of points on the original trajectory, and $N_{S}$ denotes the number of points on the simplified trajectory.

The violations denote whether the simplified trajectory intersects the boundary of the obstacle. The violations can be expressed as Equation (8).

(8) $$violations=\overset{N-1}{\underset{n=1}{\sum }}LIP\left(p_{i},p_{i+1}\right),$$

where LIP denotes Algorithm 1 *LineIntersectPoly* and $p_{i}$ is a point on the trajectory. When the line of the trajectory intersects the boundary of the obstacle, LIP is true and 1. Otherwise, LIP is false and 0. After LIP is performed on all line segments on the trajectory, violations are the summation of all the LIP results.

## 3. Results

**Figure 7:**
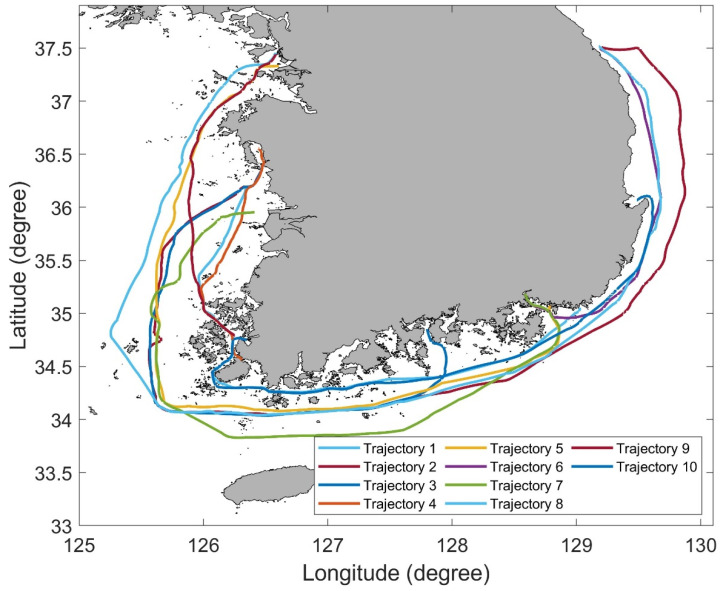
AIS trajectories in Korean sea for verifying the performance of the proposed algorithm.

To verify the effectiveness of the ODP algorithm, experiments were conducted on trajectories 1 and 2 in the Korean sea, as shown in Figure 7. The AIS trajectory set consists of 10 trajectories with diverse departure and arrival. A sensitivity analysis on the threshold (ϵ) of the ODP algorithm was conducted. In addition, we compared the performance of the ODP algorithm with that of the existing DP algorithm. Finally, the computation time was compared depending on whether the PMR quadtree was applied or not.

### 3.1. Comparison of Quadtree and Uniform Method

In the quadtree method, many nodes are generated in areas with many obstacles; otherwise, nodes are generated sparsely. Generally, the quadtree method can be compared with the uniform method. Table 1 shows the simplification using the quadtree and uniform methods for Trajectory 1. The number of edge comparisons means the number of intersections between the line segment of the obstacle and the trajectory when ODP is progressed. The resolution of the uniform method should be set to 1024, based on the minimum node size of the quadtree. The number of nodes generated by the uniform method is about 19 times more than when generated by the quadtree method. If the resolution is set to 256, a similar number of nodes can be generated. However, the number of edge comparisons increases greatly when performing ODP. Therefore, the quadtree method intensively creates nodes in areas with obstacles, which can reduce the number of edge comparisons while reducing the total number of nodes obtained from the uniform method.

### 3.2. Comparison of AIS Trajectory Simplification Results between the ODP and DP Algorithm

The ODP and DP algorithms were applied to all trajectories for comparison. Among them, trajectories 4 and 10 were selected for sensitivity analysis because the environment of these trajectories has many obstacles. Table 2 shows the results of the sensitivity analysis of the threshold for trajectory 4. In the DP algorithm, the threshold is the maximum perpendicular distance from the simplified line segment to the original trajectory. Since the maximum perpendicular distance is similar to the radius of the ship domain, the size of the ship domain can be used to determine the threshold [31]. The ship domain can be set with various sizes and shapes depending on the navigation situation. In recent studies, the ship domain was up to 1 km with a complex shape [64]. Therefore, sensitivity analysis was performed by setting the threshold up to 1 km.

**Figure 8:**
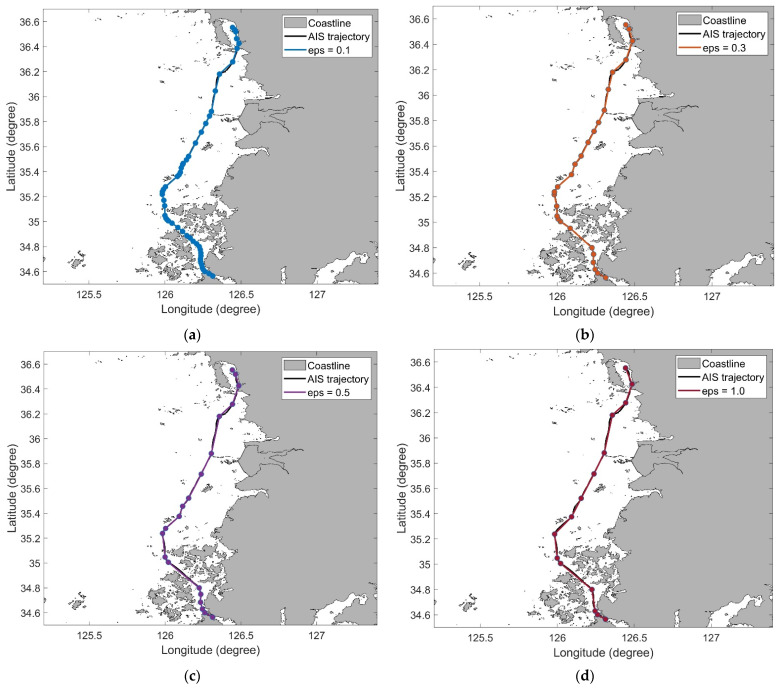
AIS trajectory simplification results of the ODP algorithm for trajectory 4: (**a**) ϵ=0.1; (**b**) ϵ=0.3; (**c**) ϵ=0.5; (**d**) ϵ=1.0.

We set thresholds for ODP and DP to 0.1, 0.3, 0.5, 0.7, and 1.0 km. The length of trajectory 4 is 242.49 km, and the number of points on trajectory 4 is 2420. In Table 2, the number of violations represents the number of times the simplified trajectory intersects the obstacle. Because the ODP algorithm is a modified DP algorithm, their simplification results were similar. As the threshold increased, several data points in trajectory 4 were removed, and consequently, the distance of the trajectory reduced. The simplified trajectories generated using the ODP algorithm are shown in Figure 8. In all the results, the intersection of the trajectory and obstacle was not observed.

**Figure 9:**
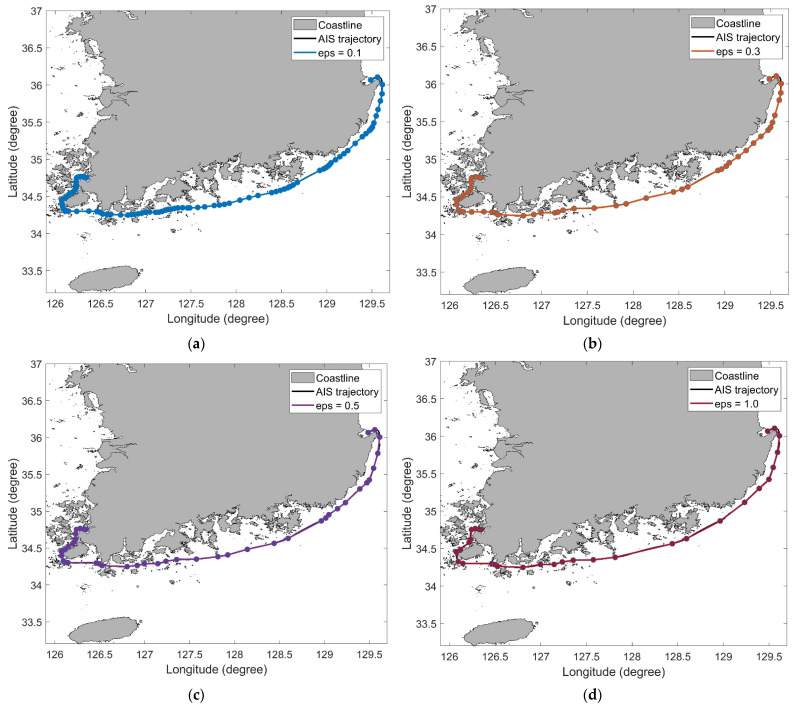
AIS trajectory simplification results of the ODP algorithm for trajectory 10: (**a**) ϵ=0.1; (**b**) ϵ=0.3; (**c**) ϵ=0.5; (**d**) ϵ=1.0.

Table 3 shows the results of the sensitivity analysis of the threshold for trajectory 10. The length of trajectory 10 is 513.06 km, and the number of points on trajectory 10 is 5122. Similar to the results for trajectory 4, it was confirmed that, as the threshold increased, several data points in trajectory 4 were removed, and thus, the distance of the trajectory also reduced. The simplified trajectories obtained using the ODP algorithm are illustrated in Figure 9, which shows no intersection of the trajectory and obstacle.

### 3.3. Comparison with Other Methods

To verify the usefulness of the ODP algorithm, it was compared with other simplification algorithms, including the DP algorithm and the algorithm considering ship behavior [46]. Table 4 shows the results of the ODP and other algorithms. In areas with many obstacles, the DP algorithm and Wei et al. [46]’s algorithm show that violations occur frequently. The ODP algorithm shows no violations in all cases. Since the ODP algorithm is a DP-based algorithm, it was confirmed that the ODP results were similar to the DP results. The compression rate of ODP was slightly higher than that of the DP algorithm because the trajectory with obstacles was not simplified. For the same reason, the length loss of ODP was slightly lower than that of the DP. In terms of length loss and compression rate, ODP was generally better than the algorithm of Wei et al. [46].

**Figure 10:**
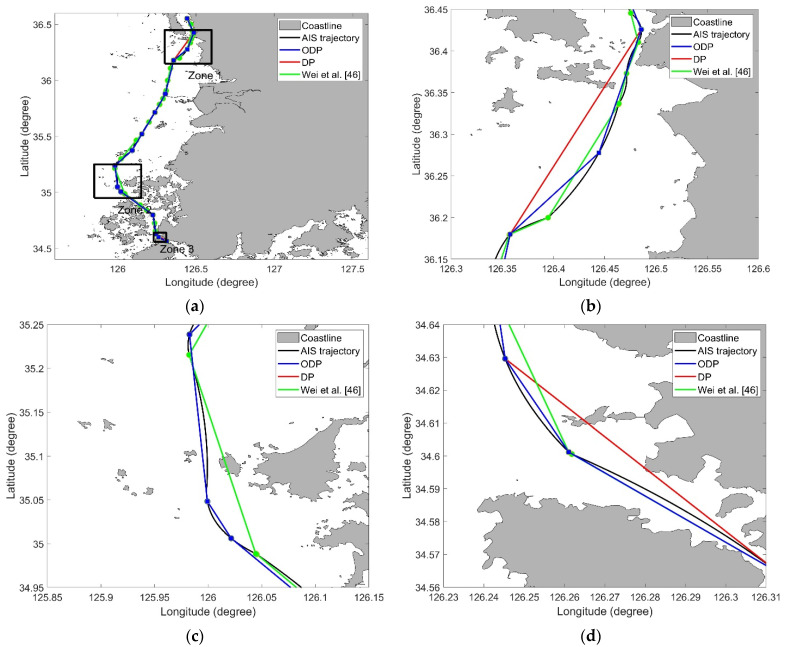
Results obtained from the ODP and DP algorithms for trajectory 4 (ϵ=1.0): (**a**) Overview; (**b**) Zone 1 in (**a**); (**c**) Zone 2 in (**a**); (**d**) Zone 3 in (**a**) [46].

Figure 10 shows the simplified trajectories generated using the ODP and DP algorithms for trajectory 4 with ϵ=1.0 km. Because the DP algorithm and the method of Wei et al. [46] remove the data points without considering the topographic information, the simplified trajectory intersects the obstacles. As can be observed from Table 4, the larger the threshold, the more violations the obstacle and trajectory cross. On the other hand, as shown in Figure 10b–d, the ODP algorithm performed trajectory simplification without the trajectory intersecting the obstacle. Since the ODP algorithm has the property of DP, it is better than the method of Wei et al. [46] in the perspective of length loss and compression rate.

**Figure 11:**
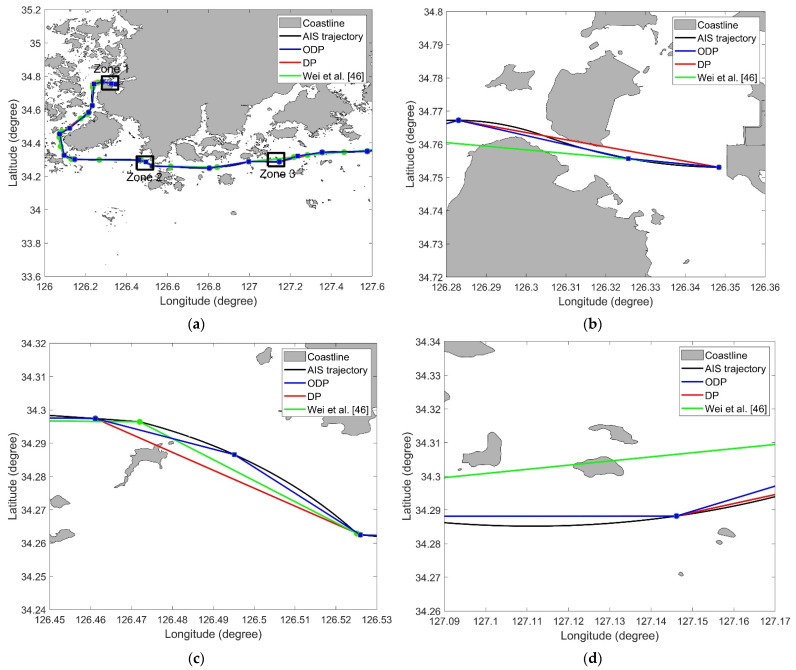
Results obtained from the ODP and DP algorithms for trajectory 2 (ϵ=1.0): (**a**) Overview; (**b**) Zone 1 in (**a**); (**c**) Zone 2 in (**a**); (**d**) Zone 3 in (**a**) [46].

Figure 11 shows the intersections of the simplified trajectory 10 and obstacles. As can be observed from Table 4, the larger the threshold, the more violations the obstacle and trajectory cross. Figure 11a–d shows the enlarged visualizations of the areas containing intersections of the trajectory and obstacle. From these results, it can be concluded that the DP algorithm and the method of Wei et al. [46] yield simplified trajectories intersecting the obstacles, and the ODP algorithm yields simplified trajectories that do not intersect with the obstacle. Similar to the results for trajectory 4, the ODP algorithm is better than the method of Wei et al. [46] from the perspective of length loss and compression rate. From these results, it can be concluded that the ODP algorithm is more practical and suitable for navigation safety.

### 3.4. Computation Efficiency of the ODP Algorithm with PMR Quadtree

Table 5, Table 6 and Table 7 show the computation times of the ODP algorithm with and without the PMR quadtree for trajectories 1, 4, and 10. A typical ILP algorithm was used in the ODP algorithm without the PMR quadtree. The computation time for each threshold presented in Table 5, Table 6 and Table 7 is the average computation time for 10 repetitions. For ϵ=1.0 of Table 5, Table 6 and Table 7, it is shown that the ODP algorithm with the PMR quadtree reduced the computation time by about 89% compared to that without; the ODP algorithm with the PMR quadtree is superior to that without. Furthermore, it can be seen that the ODP algorithm with the PMR quadtree yielded a more robust performance in terms of the computation time than that without. Therefore, we can conclude that the proposed method is worth introducing in practice because it has the most reliable performance.

## 4. Conclusions

The AIS trajectory stores ship location information based on a time series. The longer the storage time, the larger the size of the data. This renders it difficult to store and process the data. Therefore, AIS trajectories simplification plays a pivotal role in the study of maritime fields such as weather routing and safety navigation.

In this study, the ODP algorithm based on the DP algorithm was proposed. The proposed algorithm simplifies AIS trajectories through the data preprocessing that removes outliers and creates trajectories, checking the intersections of line segments and polygons using the PMR quadtree, and the DP algorithm, considering topographic information. We conducted experiments on real-world trajectories in the Korean sea to verify the performance of the proposed algorithm. The experimental results indicated that the ODP algorithm yielded simplified trajectories with no intersections of the trajectory and obstacle, unlike the DP algorithm. In addition, the ODP algorithm with the PMR quadtree was up to 30 times faster than that without the quadtree. This result suggests that the performance of the proposed algorithm is more effective in the Korean sea with complex topography.

Future studies can focus on extending our method in consideration of various information on ships. The proposed method considered only the ship location based on AIS trajectories. It is difficult to generate a simplified trajectory with a high compression ratio and low distortion when the trajectory contains the rotation due to obstacles. To solve this problem, it would be interesting to extend our method by considering the speed and direction of the ship and extracting more meaningful AIS data points.

## Figures and Tables

**Table 1:** Comparison of the results obtained using the quadtree and uniform methods (Trajectory 1).

Method	The Number of Nodes	Minimum Node Size (km)	The Number of Edge Comparisons
Quadtree	55,534	0.407	95
Uniform (256 × 256)	65,536	1.629	2534
Uniform (512 × 512)	262,144	0.814	355
Uniform (1024 × 1024)	1,048,576	0.407	95

**Table 2:** Comparison of the results obtained using the ODP and DP algorithms for trajectory 4.

	ODP Algorithm			DP Algorithm		
ϵ (km)	Length Loss (%)	Compression Rate (%)	Number of Violations	Length Loss (%)	Compression Rate (%)	Number of Violations
None	100.0	100.0	-	100.0	100.0	-
0.1	99.62	97.85	0	99.62	97.85	0
0.3	99.49	98.88	0	99.49	98.88	0
0.5	99.43	99.17	0	99.18	99.21	1
0.7	99.26	99.38	0	99.01	99.42	1
1.0	99.26	99.38	0	98.92	99.46	2

**Table 3:** Comparison of the results obtained using the ODP and DP algorithms for trajectory 10.

	ODP Algorithm			DP Algorithm		
ϵ (km)	Length Loss (%)	Compression Rate (%)	Number of Violations	Length Loss (%)	Compression Rate (%)	Number of Violations
None	100.0	100.0	-	100.0	100.0	-
0.1	99.61	98.03	0	99.61	98.03	0
0.3	99.49	98.97	0	99.49	98.98	0
0.5	99.44	99.14	0	99.44	99.16	1
0.7	99.33	99.32	0	99.3	99.36	1
1.0	99.26	99.39	0	96.85	99.51	2

**Table 4:** Results of ODP and other algorithms.

	DP			Wei et al. [46]			ODP		
Trajectory	Length Loss (%)	Compression Rate (%)	Number of Violations	Length Loss (%)	Compression Rate (%)	Number of Violations	Length Loss (%)	Compression Rate (%)	Number of Violations
1	0.74	99.48	1	1.04	98.77	3	0.72	99.47	0
2	0.71	99.62	1	0.67	98.73	1	0.46	99.60	0
3	0.98	99.61	1	0.45	98.83	1	0.56	99.59	0
4	1.08	99.46	2	1.13	98.35	2	0.74	99.38	0
5	0.40	99.56	1	0.48	98.79	1	0.37	99.55	0
6	0.58	99.51	1	0.60	97.65	1	0.55	99.48	0
7	0.46	99.50	1	0.55	98.43	2	0.45	99.48	0
8	0.38	99.57	2	0.45	99.12	1	0.36	99.55	0
9	0.64	99.51	1	0.79	99.01	1	0.61	99.48	0
10	3.15	99.51	5	0.58	98.42	2	0.74	99.39	0

**Table 5:** Comparison of the computational efficiencies of the ODP algorithms with and without the PMR quadtree (Trajectory 1).

ϵ (km)	ODP with PMR Quadtree	ODP without PMR Quadtree	Ratio (%) (with/without)
0.1	6.4	169.3	3.78
0.2	6.2	115.1	5.39
0.3	5.8	87.1	6.66
0.4	5.6	75.1	7.46
0.5	5.4	66.6	8.11
0.6	5.2	59.5	8.74
0.7	5.2	55.2	9.42
0.8	5.0	50.4	9.92
0.9	5.0	45.8	10.92
1.0	4.9	43.8	11.19

**Table 6:** Comparison of the computational efficiencies of the ODP algorithms with and without the PMR quadtree (Trajectory 4).

ϵ (km)	ODP with PMR Quadtree	ODP without PMR Quadtree	Ratio (%) (with/without)
0.1	6.9	180.1	3.83
0.2	6.4	127.7	5.01
0.3	6.0	99.4	6.04
0.4	5.8	78.6	7.38
0.5	6.0	68.3	8.78
0.6	5.4	62	8.71
0.7	5.6	55.7	10.05
0.8	5.2	51.6	10.08
0.9	5.0	47.4	10.55
1.0	5.1	46.1	11.06

**Table 7:** Comparison of the computational efficiencies of the ODP algorithms with and without the PMR quadtree (Trajectory 10).

ϵ (km)	ODP with PMR Quadtree	ODP without PMR Quadtree	Ratio (%) (with/without)
0.1	6.7	174.2	3.85
0.2	6.2	118	5.25
0.3	5.8	89.8	6.46
0.4	5.6	76.1	7.36
0.5	5.4	66.3	8.14
0.6	5.2	59.7	8.71
0.7	5.2	55.8	9.32
0.8	5.1	51.3	9.94
0.9	4.9	45.4	10.79
1.0	4.9	43.4	11.29

## Data Availability

Not applicable.
